# Control of adhesion and protrusion in cell migration by Rho GTPases

**DOI:** 10.1016/j.ceb.2018.09.003

**Published:** 2019-02

**Authors:** Harry Warner, Beverley J Wilson, Patrick T Caswell

**Affiliations:** Wellcome Trust Centre for Cell-Matrix Research, Faculty of Biology, Medicine and Health, University of Manchester, Manchester Academic Health Science Centre, Manchester, UK

## Abstract

Cell migration is a critical process that underpins a number of physiological and pathological contexts such as the correct functioning of the immune system and the spread of metastatic cancer cells. Central to this process are the Rho family of GTPases, which act as core regulators of cell migration.

Rho GTPases are molecular switches that associate with lipid membranes and act to choreograph molecular events that underpin cell migration. Specifically, these GTPases play critical roles in coordinating force generation through driving the formation of cellular protrusions as well as cell–cell and cell–matrix adhesions.

Here we provide an update on the many roles of Rho-family GTPases in coordinating protrusion and adhesion formation in the context of cell migration, as well as describing how their activity is controlled to by a variety of complex signalling networks.

**Current Opinion in Cell Biology** 2019, **56**:64–70

This review comes from a themed issue on **Cell architecture**

Edited by **Johanna Ivaska** and **Manuel Théry**

For a complete overview see the Issue and the Editorial

Available online 3rd October 2018

**https://doi.org/10.1016/j.ceb.2018.09.003**

0955-0674/© 2018 The Authors. Published by Elsevier Ltd. This is an open access article under the CC BY license (http://creativecommons.org/licenses/by/4.0/).

Rho-family GTPases are molecular switches; most which cycle from an ‘on’ GTP bound state to an ‘off’ GDP bound state, driven by GEFs (guanine nucleotide exchange factors) and GAPs (GTPase-activating proteins) respectively. Association with lipid membranes through a lipid (farnesyl or geranylgeranyl) tail ensures Rho family GTPases signal at membrane-cytosol interfaces and exquisite control the ratio of cytosolic to membrane bound GTPase is achieved by the Rho-GDI (Rho GDP-dissociation inhibitor) family of proteins [1]. An atypical subgroup of Rho-family GTPases, known as the Rnd family are constitutively GTP bound, and instead are thought to be regulated by control of their association with lipid membranes, via 14-3-3 proteins which can bind to Rnd GTPase lipid tails [2]. Through the extensive regulation of Rho GTPase activation and localisation the cell can control the activation of Rho-family GTPases in a precise spatio-temporal manner [1]. In fact Rho-family GTPases have long been appreciated as signalling molecules that allow the cell to relay information to a variety of cellular machineries including the NADPH oxidase complex and vesicle trafficking components [3,4]. The role of Rho GTPases in controlling the actin cytoskeleton was highlighted by Alan Hall’s seminal work linking RhoA, Rac1 and Cdc42 to the formation of stress fibres, lamellipodia and filopodia, respectively [5, 6, 7]. Furthermore, the discovery that RhoA drives the formation of stress fibres highlighted the importance of Rho GTPase signalling during the formation of cell–matrix adhesions [6]. This review will focus on Rho GTPase signalling in the context of cell migration, examining how these molecular switches signal to cellular protrusions and cell–matrix adhesions. Here we summarise what is known about Rho-family GTPases in the context of leading edge protrusion formation, highlighting recent studies that have helped to uncover the complexity of these fascinating molecular switches. Specifically, this review will highlight four major aspects of Rho GTPase biology: the effectors of Rho GTPases, the regulators of Rho GTPases, the role of Rho GTPases in determining cellular directionality and the importance of Rho GTPases in the context of cell–matrix adhesions. All four aspects play major roles in understanding how Rho GTPases signal during migration and all four are far from being fully understood.

## Rho-family GTPase effectors

**Figure 1:**
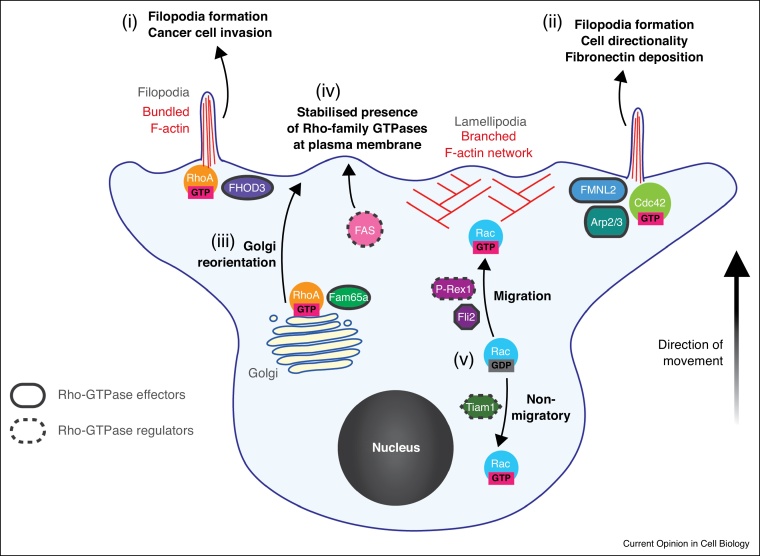
Rho GTPases in protrusion formation summary. (i) RhoA can signal to the formin FHOD3, via the ROCK family kinases, to promote the invasion of cancer cells into 3D fibronectin rich ECM. This form on invasive migration occurs downstream of the upregulated endocytic recycling of the α5β1 integrin, and does not require the action of the Arp2/3 complex. (ii) Cdc42 can drive the formation of filopodia by activating the formin FMNL2 and/or Arp2/3. (iii) RhoA-FAM65 interaction can re-orientate the Golgi apparatus towards the leading edge of the cell in 2D environments, facilitating efficient migration. (iv) Fatty acid synthesis alters the biochemical and biophysical properties of the plasma membrane, stabilising the presence of Rho GTPases in the membrane. This may have important implications for understanding how the metabolic state of a cell may affect its ability to migrate. (v) Different GEFs can promote differential Rac1 signalling, either promoting a migratory output by ensuring Rac1 binds to FLI2 (P-Rex1) or preventing a migratory output (Tiam1).

Following the discovery that Rac1 and Cdc42 stimulate the formation of lamellipodia and filopodia respectively, numerous factors were identified that enable these GTPases to build a protrusive leading edge. Of key importance are the proteins that enabled Rac1 and Cdc42 to drive actin nucleation. These included the Arp2/3 activators of the WAVE and WASP family for both Rac1 and Cdc42 respectively [8,9]. The discovery of these proteins led to the concept, based on 2D cell culture studies that Rac1 and Cdc42 signalling to the Arp2/3 complex is essential for the establishment of the leading edge. However this concept was extended and challenged by the direct observation of RhoA signalling at the leading edge of mouse fibroblasts and human cancer cells migrating in 2D cell culture [10, 11, 12, 13]. Furthermore knockout studies of Arp2/3 complex components in fibroblasts migrating in 2D demonstrated that Arp2/3 is not a universal requirement for movement on such surfaces, although defects in lamellipodia formation and directional migration in both haptotaxis and chemotaxis have been observed [14, 15, 16]. The universal requirement for Arp2/3 in migration was also challenged by the discovery of amoeboid migration which utilises RhoA signalling at the leading edge of the cell to disrupt cortical actin, allowing the cell to control the number and size of plasma membrane-based blebs that drive the cell’s movement through gaps in 3D extracellular matrix [17, 18, 19]. Therefore, it is not surprising that studies continue to identify proteins that act downstream of Rho-GTPases to facilitate protrusion formation and couple such formation to the motility of the rest of the cell. An example of such work includes the identification of FAM65A as a RhoA effector. By binding to Golgi associated FAM65A, RhoA is thought to re-orientate the Golgi network towards the leading edge, facilitating efficient migration of single cells in 2D [20]. FMNL2 has recently been identified as a formin that localises to the leading edge of cells in 2D and promotes filopodia formation downstream of Cdc42 [21]. Furthermore RhoA activation at the leading edge of cells in 3D matrix promotes filopodia formation and invasive migration through ROCK-mediated activation of the formin FHOD3 [22,23] (Figure 1).

Given the complexity of the leading edge of migrating cells and the refinement of methodologies being developed to study it, it seems likely that the list of proteins known to act down stream of Rho GTPases will continue to grow. This should ultimately provide a more in-depth understanding of migration in both physiological and pathological contexts.

## Cellular directionality

**Figure 2:**
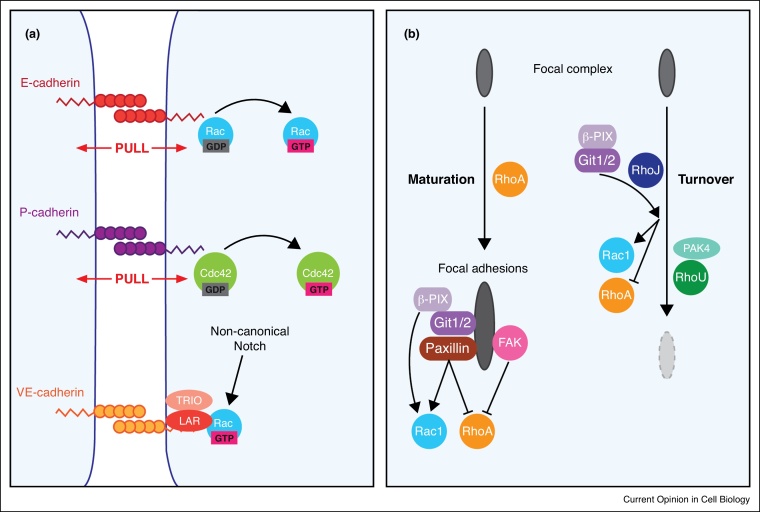
Rho family GTPases in the context of adhesion. **(a)** Rac1 and CDC42 control the directionality of groups of migrating cells when activated via the mechanical stretch of E-cadherin and P-cadherin respectively. Non-canonical notch signalling leads to the formation of a VE-Cadherin-LAR-TRIO complex that leads to the activation of Rac1. **(b)** RhoA signalling can promote focal complex maturation, leading to the recruitment of integrin associated proteins. These proteins include the Git1/2-β-Pix complex that binds to paxillin and signals to promote Rac1 signalling and supress RhoA signalling. RhoJ can enhance focal complex turnover, by recruiting the β-Pix-GIT complex in order to block RhoA signalling, blocking RhoA mediated focal adhesion maturation. RhoU, when stabilised by PAK4 can also promote the turnover of focal adhesions.

To obtain a comprehensive understanding of cell migration, it is essential to understand how cues from the cell’s external environment are relayed to the actin cytoskeleton, so the cell can migrate towards the cue; a process herein referred to as cellular directionality. Understanding cellular directionality is particularly important for the cell migration field as motile cells must be able to both prioritise external cues and rapidly change direction in response to an ever-changing external environment. Whilst the types of cue that can trigger cell migration (e.g. chemokines, matrix-derived etc.) have been well described, the search for an internal ‘compass’ has proven somewhat difficult [24,25]. Whilst for years this role was thought to be provided by PI3 kinase, the discovery that cells can migrate in the absence of this kinase re-opened this question [26, 27, 28]. Increasingly however, the Rho-family GTPases have been implicated in this role. For example Rac1 signalling can relay directional information between *Drosophila* border cells migrating as a cluster *in vivo*, via E-cadherin mediated mechano-sensing [29]. Similarly, P-cadherin mediated mechano-transduction can drive cell polarisation during collective mouse myoblast migration in a 2D culture system, by signalling to Cdc42 [30] (Figure 2a).

Cdc42 has also been implicated as an internal compass during neutrophil migration. In order to successfully trap and destroy motile bacteria, neutrophils must rapidly respond to the ever changing position of the bacterium [31]. Rho-GTPase FRET sensors, in combination with photoactivatable chemokines demonstrated a role for Cdc42 in responding to the chemokine and controlling neutrophil steering (and suppression of RhoA), whereas a shallow gradient of Rac activity more distal to the leading edge might provide the ‘engine’ [32^••^]. This neutrophil study was performed in 2D culture, and thus it remains to resolved if Cdc42 performs this role during *in vivo* migration, whereby the neutrophil must integrate and prioritise numerous migratory cues.

It is interesting to note that Rac1 and Cdc42 have both been shown to control cellular directionality: Rac1 in the collective migration of *Drosophila* border cells [29] and in fibroblasts [33] and Cdc42 in both collective cell migration and in neutrophils [32^••^,^30^]. Cdc42 would seem to be the more obvious candidate as a universal compass, should such an entity exist, given its defined role in establishing cell polarity [34,35]. However, given the significant differences between the cell types used in these studies, and the difficulty of finding a universal compass that controls the directionality of a migrating cell, it is likely that different members of the Rho family of GTPases can serve as a compass in a context-dependent manner.

## Signalling to Rho-family GTPases

Since the discovery of small GTPases, many questions have persisted as to the nature of the GEFs and GAPs that control the on/off cycle of these switches [1]. Rho-family GTPases are no exception, and despite numerous regulators having been identified, it is still not clear why there are so many or how much functional redundancy exists. Answering these questions is essential as GEFs and GAPs provide an interface through which the cell is able to communicate to Rho-family GTPases [1]. Recently, work from Marei *et al.* has addressed this question in a mammalian culture system, confirming the relevance of previous studies in yeast. In the NIH3T3 mouse embryonic fibroblast cell line, the Rac GEF P-Rex1 promoted cell migration in 2D and a more contractile phenotype in 3D, whilst TIAM1 signalled to block migration. The key to these differential outcomes seemed to be dependent on P-Rex1 enhancing the interaction of Rac1 with FLI2 [36,37,38^•^]. This work suggests that GEFs may serve to function as more than just ‘switch flippers’ and act to direct Rho GTPase signalling via specific effector pathways (Figure 1).

In addition to understanding the GEF-GAP cycle regulating Rho-family GTPases, studies have continued to address the role of the Rho-GDIs, which binds to the geranylgeranyl tail to sequester Rho-family GTPases from interacting with lipid membranes, preventing their activation. Whilst it would be tempting to speculate that Rho-GDIs act to maintain a pool of unused Rho-family GTPases, a recent study has shown that Rho-GDI serves a specific role in controlling the level of Rho-GTPase activation by coordinating GTPase activity and re-activation on a ∼10 s timescale [39]. Furthermore, a role for the lipid composition of the plasma membrane in regulating this association has been proposed. By inhibiting fatty acid synthetase (FAS) in migrating inflammatory macrophages, Wei *et al.* were able to demonstrate a role for fatty acids in stabilising the presence of Rho-family GTPases at the plasma membrane, in the context of diabetes [40^•^] (Figure 1).

Despite an in-depth understanding of the proteins that directly interact with Rho GTPases to switch them on and off, numerous questions persist as to which signalling pathways act upstream of these regulators, and where GEFs, GAPs and GDIs feature in terms of the complex signalling networks that are known to promote cell migration. Recent studies have sought to address this by both extending the list of proteins that are known to signal upstream of Rho-family GTPases, as well as utilising a variety of systems-based techniques based on mathematical modelling to predict how Rho-family GTPases will signal in response to perturbations of the signalling networks they interact with.

Rho-GTPases have long been known to signal downstream of a variety of receptors such as receptor tyrosine kinases (RTKs), G-protein-coupled receptors (GPCRs) and integrins to name a few. Recently, a study has extended this list, identifying a novel role for non-canonical Notch signalling in driving Rac1 activity via the GEF TRIO, which in turn reinforces the formation of adherens junctions [41^••^] in endothelial cells both *in vitro* and *in vivo*. It will therefore be interesting to observe if this pathway plays a role in collective cell migration, which is co-ordinated via cadherin-based adhesions (Figure 2a).

In terms of placing Rho-family GTPases within the context of a signalling network, Boolean modelling of Rac/RhoA signalling in invasive cancer cells has established a link between MAP kinase signalling downstream of RTKs, and the activation of RhoA, which we had previously shown to drive invasion into fibronectin-rich extracellular matrix [22,23]. Model simulations predicted MAPK signalling controls a negative feedback loop via the Sos1-Eps8-Abi1 complex that supresses Rac1 activity, enabling the activation of RhoA in cells migrating both in 2D plastic and 3D cell-derived matrix. Experimental inhibition of MAP kinase signalling enabled the re-activation of Rac1 at the leading edge of the cell, supressing filopodia formation and invasion into extracellular matrix and on cell-derived matrix. Critically, knockdown of Eps8 (a key component of the RacGEF complex in this system) rendered cells insensitive to MAPK inhibition, re-enabling cells to activate RhoA at the leading edge of the cell, driving invasive migration [42^•^]. Such feedback loops may provide plasticity to the migrating cell, enabling it to re-programme its leading edge in response to a changes in the surrounding environment [43].

Similar approaches using more sophisticated kinetic modelling identified a role for PAK signalling in mediating a bi-stable switch [44]. Exposing MDA-MB-231 breast carcinoma cells in 2D culture to increasing amounts of PAK inhibitor had different effects on Rac and RhoA signalling depending on whether cells had been pre-incubated with the same inhibitor, demonstrating the predicted hysteresis. Interestingly this bi-stability is conserved in actin dynamics, and suggests that cytoskeletal signalling pathways encode a memory of activation status [44].

In summary, it is becoming increasingly clear that as the list of Rho GTPase regulators increases, systems-based studies are needed to understand how these regulators function as a network. Furthermore, mathematical modelling enables the development of unique hypotheses that cannot be generated through qualitative analysis, and produces specific, testable predictions. Therefore, it is apparent that there is much to be learnt about Rho-family GTPases through quantitative mathematical modelling.

## Rho GTPases in cell–matrix interactions

The importance of Rho-family GTPases in cell matrix interactions has been well appreciated ever since the initial identification of RhoA as a regulator of stress fibres, which showed that focal adhesions are unable to form in the absence of RhoA signalling [6]. Since then, a number of studies have shown extensive reciprocal signalling between matrix receptors and Rho-family GTPases, however for the purpose of this review, we shall focus on a handful of recent studies that have extended our understanding of direct signalling between focal adhesions and Rho GTPases.

Focal adhesions have long been known to control the activity of Rho-family GTPases via adaptor proteins that can signal to GEFs and GAPS, such as paxillin, which can signal to both activate Rac1 and suppress RhoA, and FAK which can signal to supress RhoA activity [45,46]. β-Pix is a Rac GEF recruited to adhesion complexes through interaction with Git1/2 recruitment to paxillin [47,48]. Interestingly Git1/2-β -Pix can also be recruited to adhesion complexes by RhoJ, which mediates adhesion turnover by sustaining Rac1 activity and preventing RhoA activation [49]. These types of interactions can govern the transition of nascent adhesion complex to focal complexes, but restrain the maturation to focal adhesion (which requires RhoA–driven contractility [6,50]). Interestingly, RhoU is stabilised by interaction with PAK4 in a Cdc42 and kinase-independent manner to regulate adhesion turnover [51]. This suggests that complex feedback networks exist between Rho GTPases and adhesion complexes that might determine the intricate and subtle morphological adaptations of adhering and migrating cells (Figure 2b). All these studies were performed principally in 2D cell culture and thus it remains to be understood how RhoJ and RhoU mediate crosstalk with focal complexes in 3D matrix environments.

Whilst it has long been appreciated that Rho-family GTPases are able to signal directly to focal adhesions, it is also becoming increasingly clear that they can achieve this indirectly, through their influence on the extracellular matrix to which the integrins bind. Cdc42 and RhoA have long been known to promote the trafficking of metalloproteinases to the tips of invadopodia to promote cancer cell metastasis by driving an interaction between IQGAP with the exocyst complex [52]. More recently a study has established a clear link between Cdc42 and fibronectin deposition allowing for the formation of focal adhesions within lamellipodia and permitting migration over the resulting matrix in a Rac1-dependent manner [53^•^]. Given that filopodia drive cancer cell invasion into fibronectin containing matrix [23,54], it will be fascinating to discover if filopodia can also drive fibronectin deposition *in vivo.*

Whilst cell–matrix interactions are relatively well understood in 2D, it is vital that these studies are translated to 3D *in vivo* systems given the stark biochemical and mechanical differences between such systems. Understanding how Rho-family GTPases coordinate cell–matrix interactions *in vivo* is particularly challenging given the technical difficulties that are associated with studying cell–matrix receptors, such as integrins, in 3D. However, studying how Rho-GTPases coordinate cell matrix interactions *in vivo* is essential to understanding cell migration, in contexts such as wound healing and cancer.

## Conclusion

The complexity and intricacy of Rho-family GTPase signalling continues to increase as methodologies for studying them becomes more advanced. It is becoming clear that the plasticity and variety of structures that can be found at the leading edge is huge and more work is required to understand how Rho-GTPases signal. This will involve the identification of more Rho GTPase binding partners and an increased systems level understanding of their function that incorporates features of the extracellular environment. Furthermore, the diversity of contexts in which this family of proteins have been studied is large, and the results vary accordingly, highlighting the need to understand how Rho family GTPases and their associated proteins evolved to meet the varying requirements of different organisms.

## Conflict of interest statement

Nothing declared.

## References and recommended reading

Papers of particular interest, published within the period of review, have been highlighted as:

• of special interest
•• of outstanding interest
